# Addressing the health workforce crisis in Poland from the key stakeholders’ perspectives – a qualitative study

**DOI:** 10.1186/s12913-025-13150-5

**Published:** 2025-08-22

**Authors:** Kamila Michalska, Alicja Domagała

**Affiliations:** 1 Institute of Public Health, Faculty of Health Sciences, Jagiellonian University Medical College, Krakow, Poland; 2 Doctoral School of Medical and Health Sciences, Jagiellonian University Medical College, Krakow, Poland

**Keywords:** Medical staff shortages, Health workforce deficits, Healthcare policy, Human resources for health

## Abstract

**Background:**

There is an alarming shortage of medical personnel in Poland, which seriously affects the healthcare system and access to medical services. This study aims to identify possible actions aimed at mitigating and preventing staff shortages in Poland.

**Methods:**

A qualitative study was conducted as semi-structured in-depth interviews with 15 key stakeholders of the Polish healthcare system. Interviews were conducted from April to July 2024 via the MS Teams platform. A directed content analysis of the interviews was conducted using NVivo software.

**Results:**

Respondents indicated the need to develop and implement a long-term plan for the functioning of the healthcare system and medical personnel, which takes into account technological development, demographic and generational changes. Interprofessional cooperation, improved financing and working conditions, better management of available resources and attention to mental health, and implementation of activities aimed at preventing further employee outflow and burnout are necessary. It is also desirable to promote professions and educate from the youngest age - transparently showing and encouraging graduates to take up medical professions.

**Conclusions:**

Possible actions to implement, aimed at reducing and preventing the shortage of medical staff in Poland, include preparing and implementing a long-term plan based on reliable data. The involvement of various entities should be enforced, taking into account management, financing and education. In addition, desirable actions concern the analysis of the needs of employees and the difficulties they face at work.

**Supplementary Information:**

The online version contains supplementary material available at 10.1186/s12913-025-13150-5.

## Highlights

Wide, long-term plan is vital for Polish healthcare workforce sustainability.

Mental health support is critical to stop the outflow of health workers.

Investing in education and promotion of health professions is needed.

IT solutions and improved working conditions may reduce the workforce shortage.

**Supplementary Information:**

The online version contains supplementary material available at 10.1186/s12913-025-13150-5.

## Background

Healthcare systems around the world are facing many challenges and crises. The most pressing and frequently raised issue is the significant shortage of healthcare workers [1, 2]. The 2016 Global Health Human Resources Strategy: Workforce 2030 projected a global shortage of 18 million health workers by 2030 [1]. Estimates of the projected shortage by 2030 have been revised based on the 2020 Health Resources Assessment and researchers have indicated that the global shortfall by 2030 will be 10 million health workers. This is based on the information that in 2020, the global health workforce was 65.1 million and is projected to grow to 84 million by 2030, (29% growth), which is faster than population growth (9.7%) [3]. However, when it comes to the European Union, the estimated deficit of medical and care workers has increased from 1.6 million in 2013 to 4.1 million by 2030 [4]. This estimate reflects multiple factors, including demographic shifts - an aging population leading to increased healthcare demand, current workforce attrition rates due to retirements and migration, and the capacity of education and training systems to supply new healthcare professionals [1, 4].

Poland is experiencing a significant shortage of healthcare professionals and has one of the lowest health workforce indicators among the EU countries, with 3.5 doctors and 5.7 nurses per 1,000 inhabitants, compared with the EU27 average of 4.2 and 8.4, respectively [5]. The alarming shortage of medical personnel, has serious consequences for the healthcare system and the quality and availability of medical services [6]. The Department of Analysis and Strategy of the Ministry of Health (MoH) in Poland has developed a demand-supply model that takes into account gender, working time, socio-demographic changes, and according to this analysis, the demand and supply lines for doctors will intersect this year (2025). However, after 2030, the demand for doctors has been set at 144,000, and the number of doctors at the same point on the timeline will be 169,000. These models will have to be modified to take into account new data, but the problem has not been solved, because the analysis of the MoH shows that the shortages concern medical staff in specific specializations and regions. Another problem in the Polish health care system is the high average age of medical workers, in 2023 for doctors it was 50.8 years, and for nurses 49.9 years [7]. The share of medical staff of retirement age in Poland in 2023 was as much as 25%, and nursing staff 22%. According to the Maps of health needs in 2025, the difference between the number of nursing staff entering the market and the number of staff reaching retirement age is −1756.61, while for doctors it amounts to 2103.73 [7].

As the authors of the publication “Moving from a crisis of health care workers to a success of health care workers: time for action” emphasize, current health workforce crisis is multifaced, involving challenges related to staffing, training, gender equality and financing [8]. The challenge is not only retaining existing employees and recruiting new ones, but also an aging population and growing health needs [3, 4, 8, 9]. Moreover, healthcare workers face problems such as high levels of stress, excessive workload and lack of satisfaction, which may lead to burnout, and resignation from the profession [4, 8–14]. Due to the development of new technologies, as well as the entry of young generations into the labour market, it is necessary to adapt education and training to changing expectations, health needs and demand [8, 15–18]. Not only technological progress is noticeable, but also an increase in the number of working women, who constitute 3/4 of employees in European healthcare systems [19]. Women, also in healthcare sector, earn less than men, are exposed to sexual harassment, are less respected, and are less likely to achieve managerial positions [20]. This contributes to job dissatisfaction, burnout and does not encourage graduates to work in the medical sector [19–21]. Addressing this crisis requires global approach, exploring policy recommendations and improving management at many levels of the healthcare system [18]. Attention is also paid to skill-mix [22, 23], efforts based on cooperation and individual needs of health workers, ensuring their physical and mental well-being [13].

The shortage of medical personnel in Poland is driven by key systemic factors, including the aging of the workforce, regional disparities, excessive workload, disparities in wages, and a high level of administrative burden [5]. Additionally, the lack of coherent and long-term planning in the education system and underfunding of the healthcare system also contribute to this situation [5, 6]. The lack of a sufficient number of medical professionals leads to an overload of employees, longer waiting times for treatment and deterioration of patient care [24]. Thus, there is an urgent need for a comprehensive assessment of possible measures and actions that can contribute to the effective mitigation of medical staffing deficits [6, 24].

So far, there has been no research in Poland in this area that includes the positions and opinions of key health sector stakeholders. The studies conducted so far have indicated the fact of medical staff shortages, but none of them have provided detailed information on the methods and actions used and that can be implemented to counteract and mitigate the effects of medical staff shortages in Poland. This study would be an important endeavor to fill information gaps in this area and to plan actions to improve the functioning of the Polish healthcare system.

Our study aimed to identify possible actions to mitigate and prevent the health workforce (HWF) shortages in Poland. Additional goals of this study were to explore the current difficulties faced by employees in the healthcare system, examples from other countries and the challenges our system is currently facing, as well as what challenges await it in the future and how health policy should be implemented. To obtain information on these issues key stakeholders of the healthcare system were selected for an empirical, qualitative study based on in-depth interview methodology. Both the analysis of these examples from different countries and the solutions proposed by our respondents, as well as the difficulties and challenges indicated, may constitute a good source of knowledge and a basis for the analysis of medical personnel policies also for other countries.

## Materials and methods

### Data collection and analysis

A qualitative study was conducted through in-depth, semi-structured interviews using an interview guide (Appendix 1). This method was chosen because it allows for a comprehensive exploration of complex and context-dependent issues such as healthcare workforce shortages. This approach allows for the collection of detailed, nuanced insights from key stakeholders who have first-hand experience and strategic perspectives on the challenges facing the healthcare system. By involving 15 carefully selected representatives from different key institutions of the Polish healthcare system, it was possible to capture diverse perspectives and identify practical solutions at a systemic level that may not emerge using quantitative methods alone. Notably, the participant group was purposefully composed to ensure strategic and professional diversity, including national and regional health policymakers, hospital directors, representatives of professional medical chambers, and clinicians with managerial experience—individuals whose theoretical expertise and real-world roles directly influence workforce policy and implementation.

Although data saturation was achieved after 12. interview, all 15 interviews were conducted as originally planned and in accordance with the approved study protocol. This was intended to enhance analytical depth and to prevent the risk of premature or false saturation, particularly given the strategic importance and heterogeneity of our participant group. This is consistent with best practices in qualitative research, which recommend continuing data collection beyond saturation for greater sensitivity and rigor [25–27]. The use of a semi-structured interview guide provided consistency while allowing for flexibility to explore relevant themes raised by participants in more detail. To ensure the high quality of the focused content analysis [28] and to establish that data were collected, analyzed, and reported in accordance with the protocol, we used the Consolidated Criteria for Reporting Qualitative Research (COREQ) developed by Tong et al. (Appendix 2) [29]. The selection of participants for the qualitative study was purposive sampling [30], to ensure the inclusion of individuals with in-depth knowledge and experience relevant to the healthcare workforce in Poland. Participants were invited through email and/or telephone contact and informed of the purpose and scientific nature of the study. All individuals who were invited to participate accepted the invitation and took part in the interviews. Participation in the study was voluntary and all participants provided their written consent to participate in this study by sending a signed consent scan by e-mail before the interview. Respondents were selected for interview based on their knowledge/experience of the topic and due to their function - individuals were selected who are key decision makers who have a real influence on decisions made in the field involving the topic of healthcare workforce (planning, employment, financing, management, etc.). The interviews were conducted using the MS Teams platform from April until July 2024 and were carried out in the Polish language. They were subsequently translated into English by the interviewer, who is fluent in both languages and possesses a deep understanding of the study context, ensuring both linguistic precision and contextual accuracy.

The same set of 10 open-ended questions were asked to the study participants. Depending on the respondents’ answers, they were asked in the same or a different order. The average length of the interview was about an hour, the shortest lasting 32 min, and the longest 89 min. The interviews were recorded and transcribed verbatim. The interview texts were carefully read and checked by one of the researchers, and then they were coded and analyzed using NVivo software. All two researchers participated in data analysis.

The study used a combined approach to coding the data - deductively starting with a set of codes based on the research questions, and then inductively based on the content of the interviews, new codes and sub-codes were added while reviewing the data. In vivo coding was used during the data coding process, meaning that the researchers tried to use the participant’s spoken language and stay as close to the participant’s intention and meaning as possible. Simultaneous coding was also used - some codes are repeated in different thematic groups because they simultaneously code multiple categories.

## Results

### Participants

The study group consisted of 15 respondents, including seven women and eight men who are key decision-makers (managers, board members) with real influence on decisions made in the Polish healthcare system. Respondents were representatives of the Ministry of Health, the National Health Fund, local government bodies, hospital managers, and representatives of the chambers of particular medical professions (doctors, nurses, pharmacists, physiotherapists, laboratory diagnosticians) and the association of paramedics. The characteristics of the respondents are shown in Table 1. In order to ensure the anonymity of the respondents and to prevent their identification, the table provides only their profession, without indicating their position in specific institutions.

**Table 1:** Interviewee’s characteristics

Participant	Gender	Age group	Organisation	Profession
1.	female	51–60	Regional Medical Chamber	Physician
2.	female	41–50	Supreme Chamber of Nurses and Midwives	Nurse
3.	male	61–70	European Commission	Physician
4.	male	71–80	Voivodship Office	Physician
5.	male	31–40	College of Young Family Doctors	Physician
6.	male	21–30	Residents’ Agreement of the National Trade Union of Physicians	Physician
7.	female	41–50	Regional Chamber of Nurses and Midwives	Nurse
8.	female	41–50	Medical Analysis Laboratory in University Hospital	Laboratory Diagnostician
9.	male	51–60	National Chamber of Physiotherapists	Physiotherapist
10.	male	41–50	Department of Emergency Medicine at Medical College	Paramedic
11.	female	41–50	Voivodship Branch of the National Health Fund (NFZ)	Lawyer
12.	female	41–50	Regional Pharmacy Council	Pharmacist
13.	male	41–50	Orthopedic and Rehabilitation Hospital	Paramedic and Public Health Specialist
14.	male	51–60	Regional Hospital	Lawyer and Economist
15.	female	31–40	Ministry of Health	Public Health Specialist

Source: Authors’ own work

Eight men and seven women participated in the study. As many as 1/3 respondents were doctors, two nurses, a physiotherapist, a pharmacist, two paramedics, two lawyers, a laboratory diagnostician, and a public health specialist. They were all management representatives who were involved in the planning and development of the Polish health workforce. Seventeen codes and eight sub-codes were created, and after analyzing and identifying common threads reviewing the data from the analysis, six main thematic groups were formed, through which the results were presented. Table 2 contains the information about the codes, subcodes, thematic groups and categories.

**Table 2:** Thematic groups, codes and subcodes

Thematic groups	Categories	Codes (subcodes)
1. Assessment of current healthcare workforce shortages and difficulties related to the specific professions	Assessment of HWF shortages	Assessment of HWF shortages Competences Data Difficulties and barriers Remuneration Education and training (Campaigns, promotion and health education, Quality of education, Specialisations) Policies and regulations (employment policies)
	Difficulties and barriers related to the specific profession	
	Perspective and data credibility	
2. Current and proposed solutions	Current solutions	Solutions (current solutions, proposed solutions, IT solutions) Policies and regulations (employment policies, migration regulations)
	Proposed solutions	
	IT Solutions	
3. Challenges for the healthcare system	Challenges	Challenges Migration Policies and regulations (employment policies, migration regulations) Private sector
4. Examples and good practices identified in other countries	Education and recognition of competences	Examples from other countries Financing Education and training (Campaigns, promotion and health education, Quality of education, Specialisations) Non-financial benefits or motivators
	Policies and programmes	
5. Generational change	New generations	Education and training (specialisations) Non-financial benefits or motivators
	The choice of specialisation	
	Work-life-balance	
6. Consequences and impact on healthcare system	Lower quality of education	Education and training (quality of education, specialisations) Financing Migration Consequences Impact on Healthcare System Policies and regulations (employment policies, migration regulations)
	Resignation from practicing a profession or migration	
	Unsuitable employees	
	Immigration of workers from Ukraine	

Source: Authors’ own work

### Assessment of current healthcare workforce shortages and difficulties related to the specific professions

According to our respondents, there is a shortage of medical staff and it is noticeable in everyday work. Sometimes it applies only to a selected group of medical professionals, a specific specialization or a specific region.

*“There is no such specialization in which there would be no deficit […] or occurs the excess of physicians.”[R#4]*

*“A large percentage of hospitals in Poland suffer from shortages of nursing staff and have difficulties in ensuring minimum employment standards for nurses.” [R#7]*

*“Doctors do not have time to talk to patients about their drug therapy.” [R#12]*

In addition to deficiencies, which have a huge impact on the quality of work performed, respondents indicated that there are additional difficulties in the workplace that influence the occurrence and deepening of shortages. These include: non-compliance with employment and remuneration standards, work organization and management not meeting the needs of employees, too much paperwork and bureaucracy, little respect, as well as excessive workload, on-call and night shifts, high level of stress and responsibility, and lack of work-life-balance due to unsatisfactory earnings and the need to combine many positions in different facilities.

*“Medical staff in Poland is very poorly managed.” [R#6]*

*“We also have a problem with bureaucracy and certain legal regulations*,* and if it was regulated*,* it would also be more encouraging.” [R#5]*

*“People would demand that work ends at 3 p.m. and not last 24 or 36 hours. I started today’s duty*,* which will last 48 hours. Which also has a very big impact on my family life.” [R#10]*

*“[…] employees are leaving to take up work in entities where the remuneration is higher.” [R#7]*

Participants in our study also emphasize that during the analysis of the occurrence of medical staff shortages, we should not only look at numbers, but at a broader picture, through the prism of various aspects, such as geographical distribution, access to services and waiting times, age of medical staff, access to specializations.

*“Our average number of doctors is close to the European average*,* i.e. approximately 3*,*5 per 1*,*000 inhabitants*,* where the European average is approximately 4.1. Our average headcount is not as dramatic as we thought it was some time ago. Even though exceeding this number is not a satisfactory answer for someone looking for healthcare*,* the staffing deficit and any staffing policy in general should be viewed from the perspective of patient access to health care.” [R#6]*

However, often comprehensive assessment of deficits is impossible or difficult to perform due to deficiency, inconsistency or lack of reliable data.

*“In Poland the different parties do not agree to the description of the situation*,* that the data that we have - both Poland or the European Commission on the situation of the staff in Poland is largely untrue. We need to think about whether we are operating with the same data. Well*,* if it is so that “opinion leaders” are undermining the kind of data that the Polish MoH*,* or the European Commission*,* or the Central Statistical Office operates*,* well*,* we have the problem.” [R#3]*

Some interview participants pointed out that the competences and potential of their professional group are not fully used and therefore there are deficits that could be reduced. Additionally, with increased responsibility and a broader scope of competences, there should be increased remuneration.

*“There is definitely a deficit and I assess it specifically from the pharmacist’s point of view. As pharmacists we are quite a large professional group that has untapped potential. […] Doctors*,* specialists or general practitioners do not have time to talk to patients about their drug therapy.” [R#12]*

*“An expanded scope of professional competences should be reflected in proportionally higher remuneration.” [R#10]*

### Current and proposed solutions to alleviate medical staff shortages

The solution most frequently mentioned by respondents to eliminate the shortage of medical staff is increasing admission limits for medical studies and opening new medical faculties at non-medical universities. Nevertheless, the vast majority of respondents are quite critical of this solution, arguing that by increasing the number of students, the quality of education will decrease.

*“We are trying to fix it somehow by increasing recruitment to medical universities*,* but this is a very bad way*,* because increasing the number of recruitments must result in a lower level of education.”[R#4]*

*“The simplest mechanism*,* which not only Poland applies*,* well it is an increase in the number of educated students.”[R#3]*

*“The government’s programmes that we produce a massive amount of graduates is not a good solution.”[R#10]*

Currently, methods such as scholarships, providing accommodation and other additional financial and non-financial benefits are also used. However, these are not practices implemented on a national scale, but regional and local activities.

*“We have communities that are trying to attract doctors*,* offering e.g. places to live or better working conditions.” [R#3]*

Another solution that has already been implemented are legally defined minimum salaries for medical workers and the remuneration of various groups of medical professionals has already been increased many times. Some respondents, however, indicate that this increase was small compared to inflation and that earnings in other fields have also increased and are currently more attractive than in the medical sector.

*“The law on minimum wages in healthcare*,* and indeed this is quite a bit of progress. Year by year*,* we got a raise*,* I thought it was so much*,* but in fact they didn’t give a lot of money. And they’re up to 12%*,* which seemed like a lot a year. But what about how inflation in the 22nd year was 14%*,* in 2023 − 11.4% […]. [R#8]*

Respondents indicated that increasing salaries is important when it comes to eliminating the shortage of medical professionals, but it is not the only and most important aspect.

*“The shortage of medical staff results from the low level of employee remuneration*,* which makes work unattractive for paramedics. High exposure to stress*,* working at night and working on holidays is also not attractive for paramedics. […] There are no opportunities for professional development. […] And this makes people really run away from the profession and it stops being a motivation for them. [R#10]*

In addition, providing better working conditions, development opportunities, using competences, being appreciated and respected at work are other factors that, according to study participants should be taken into account in the fight against shortages.

*“The key would be to make sure that in these worst places*,* it wouldn’t be the worst. That is why I pay attention to this funding*,* I pay attention to working conditions*,* I pay attention to this approach to adverse events*,* to mental health of the staff*,* to burnout at work. The main solutions that will make it just that the efficiency of the staff will be higher*,* it will be more willing to stay in the most critical locations. There will be less burnout in these critical places.” [R#6]*

*“Creating better and better conditions. The fact that now […] specializations for physiotherapists will be free of charge […]. This will certainly increase the number of people willing to specialize.” [R#9]*

In Poland, the primary care physician plays an important role, which is why among the proposed solutions presented by our respondents there were voices that their role should be further strengthened. Recently, coordinated primary care has been introduced and is becoming increasingly common, which allows for a wider range of patient care in outpatient facilities, without the need for specialist visits. In connection with the increase in the scope of services provided, it is also necessary to constantly train employees so that they can offer patients and perform available tests without generating additional visits at a higher level - specialist or in hospital care.

*“It’s worth increasing the competences of family doctors*,* which is now happening even in coordinated care. This could also cause more doctors to choose this path.” [R#5]*

Respondents also indicated the importance of patient education, which is already being implemented by selected professional chambers, but still requires development and reaching the population on a wider scale.

*“We can have a million prevention programs*,* but people have to want to use them. Therefore*,* every good project in this field must be thoroughly thought out and analyzed.” [R#6]*

The education of patients aims to bring medical professions closer, but also to promote pro-health behaviors, prevent complications, bad habits, and thus reduce health needs and, as a consequence reduce demand for medical staff.

*“When it comes to health education*,* we need to get to the basic problem*,* the basic issue*,* that society should take action and make all decisions*,* based on healthy practices. So that society gets sick as little as possible. If society is healthier and more aware. […] We could keep or use the medical base that we have*,* so that it would meet these epidemiological needs.” [R#13]*

Moreover, according to the participants’ statements, many technological solutions, IT and AI solutions, remotely issued prescriptions, e-referrals, telemedicine and remote description of radiological examinations, as well as robots have already been implemented and should be further developed - all these elements contribute to reducing staff shortages, accelerating the provision of services and providing easier access for patients. At the same time, appropriate regulations should be introduced to ensure the safety of patients and healthcare workers.

*“We are at the current stage of technological development that allows the introduction of telemedicine. Especially in those hard-to-reach places in Poland*,* there is no easy access to specialists*,* such as those who can also be offered via telemedicine.”[R#1]*

*“These electronic sick leave certificates are really written faster. The issue of various forms*,* also applications to the sanatorium.” [R#5]*

*“We have robots in pharmacies. You don’t have to look for medicines*,* the robots do it themselves. They cost*,* but they pay for themselves very quickly. The area of ​​the pharmacy doesn’t necessarily have to be that big*,* because robots often work in the basement*,* they don’t even need light*,* so this means that the profession of pharmacist will also evolve.” [R#3]*

*“Technologies should be developed*,* they should be popularized*,* and there should be education in this area. In a situation where new systems appear*,* they should be reimbursed by the National Health Fund as quickly as possible*,* due to the fact that they limit complications and improve access to the patient*,* but it should also be well regulated so as to ensure the safety of using these technologies.” [R#6]*

IT tools and solutions are also used in data analysis and preparation of applications and programs aimed at researching the Polish market of medical students and graduates, creating a supply-demand model, and thus contributing to the improvement of health policy planning. Thanks to the development of analytical research in healthcare, it is possible to obtain data and information that indicate possible directions of changes and provide support in politicians’ decisions.

*“From the point of view of analytical work*,* we use various solutions to prepare data*,* which later feed the application and also in the next step to prepare visualizations.” [R#15]*

### Examples of actions identified in other countries

Respondents, pointing to examples of actions aimed at mitigating the effects of medical staff shortages, mentioned, among others: a different work organization, i.e. a doctor is not constantly present with the patient but is available by phone, nurses and other employees have greater competences and a wider list of possible duties, the specialization path is clearly defined and is associated with additional gratifications, the patient’s respect for medical professions is greater, and selected fields of study or medical courses are conducted in a different, shorter form with more practice, e.g. under the supervision of an experienced employee, where the graduate receives a lot of support at the beginning of his/her medical career.

*“In the United States or Switzerland or in Scandinavia*,* such a small transfer of competences*,* but also strengthening the competences of some groups in the field of health care and relieving the workload of other professionals. This is the case with nurses. In the West*,* they actually have more competences*,* they earn more and they relieve the burden on doctors.” [R#8]*

*“The system of such an internship*,* a bit like medicine in Anglo-Saxon countries in general*,* that there is a process of so-called learning by doing - I am already in the hospital and I have my own tutors who guide me through the education process among already real patients.” [R#10]*

*“More specialization in advanced life-saving procedures at the intensive care level*,* as is the case in the United States*,* where a critical care paramedic or a senior paramedic is the top of the ladder of advancement of rescuers. Who*,* in addition to having an extended scope of competences*,* also earn more. In Poland it looks like this: whether I am a good rescuer or a bad rescuer*,* I am rewarded and appreciated by my employers in the same way.” [R#10]*

In addition, respondents indicated greater awareness of patients who know where and what services they can use, what competences individual medical specialists have, but also a greater number of educational and preventive programs.

*“In England patients already know that they can come and take advantage of the knowledge of the Master of Pharmacy and get from him advice. In Poland an educational program would be necessary. It can be said that a pharmacist plays the role of a general practitioner. Besides*,* in case of emergency*,* it’s true*,* the patient comes to the pharmacy.” [R#12]*

Another example mentioned by respondents is mandatory rehabilitation for selected groups of patients. These activities positively affect patients’ well-being, fewer complications and, consequently, lower health needs, reduced use of medical services, and less frequent complications that are difficult to treat and require specialized care.

*“If we take the French model*,* there are issues of amputation*,* but also mandatory rehabilitation and physiotherapy during the postpartum period and after pregnancy. […] British physiotherapists from the orthopedic industry*,* also perform intra-articular injections*,* so as competences develop and improve*,* Western European countries also provide greater competences to physiotherapists.” [R#9]*

An important aspect noticed by the interview participants is the return of women to their professions after maternity or parental leave. There are foreign programs and interventions supporting employee retention by enabling the combination of work and family responsibilities, e.g. shorter shifts, additional days off, nursery or kindergarten located close to a medical facility.

*“This is about retention*,* which is how to encourage women who have decided to spend time with the family to return to their profession. […] These are examples in Belgium*,* or in France*,* where such programs were created*,* or in the UK.” [R#3]*

### Key challenges for the healthcare system

According to our respondents, the greatest challenge to overcome the shortage of medical staff will be developing a long-term plan based on reliable data, prepared and implemented by experts representing various institutions and sectors. Accessing, collecting and analyzing data on medical staff and their shortages is also quite a task, taking into account various specializations, geographical distribution, work in many facilities, part-time work hours and the provision of medical services by older staff with the right to retire - it is not possible to determine how long such people will work.

*“These measures must be multidimensional*,* they must be monitored*,* they must be long-term*,* they must look at these elements*,* let us say to each other in the society that is emerging*,* that is technological development*,* the ageing of the population. Also*,* the aging of human resources*,* the length of professional work*,* the limitation of the burnout*,* the work-life-balance we have to take*,* calculate it.” [R#3]*

*“The biggest challenge generally in creating these actions and tools would be the plan. The diagnosis of the current state*,* where it is measurable and the preparation of the plan including its verification and evaluation in a specific timeframe. It is necessary to lean on this long-term perspective and not just some ad hoc perspective. […] An assessment from several different perspectives - the patient’s perspective*,* the MoH and the financial perspective […] a plan must be based on the data that will answer how this system should look like in 5*,* 10*,* 15 years.” [R#11]*

Another challenge for our system is unnecessary medical procedures, ineffective tests and avoidable misdiagnoses. Therefore, reforms are necessary to support the selection of the most effective choices and treatment paths for the system and the patient.

*“We probably need to introduce a model of treatment and receive funds for effective treatment. Because this also forces in-depth diagnostics*,* making the right decision*,* applying the right treatment and if everything works out*,* then the patient should theoretically be healthy.” [R#13]*

A plan for retention, recruitment, education, funding and accountability should be developed, implemented, evaluated and continuously improved. When it comes to increasing study admission limits and creating new faculties at non-medical universities, providing staff - academic teachers, but also appropriate infrastructure for learning and practice will certainly be a huge challenge.

*“Increase in the number of medical students in training was sold as a medium*,* and because of the way it was introduced*,* the pace of introduction*,* the lack of any long-term analysis*,* it turns out that it will have more and more problems. I am concerned about the short-sightedness of the regulations introduced. That they will not be well thought out*,* there will be very narrow areas.[…] We can hire more staff*,* but this staff has to be managed well. We can add more resources*,* but the resources need to be better managed.” [R#6]*

And due to the increasing use of new technologies, IT and artificial intelligence - not only training will be needed, but also new guidelines and a clear legal framework.

*“Artificial intelligence will describe the pictures*,* but there is a question of responsibility in the event of error or confusion. We need to look at the legal aspects. When there are complications*,* there will be a search for a guilty one. It is still to be implemented*,* a responsibility*,* perhaps the consent of the patient to make artificial intelligence an active participant in his treatment. I think it’s inevitable.” [R#9]*

Moreover, the respondents point out the need to promote healthcare professions, the challenge is to encourage young people to choose a medical career path, but they take into account the fact that these professions should be presented realistically so that later there is no disappointment and resignation from the chosen path because it is not consistent with imaginations.

*“The challenge is to raise money for the promotion of the profession*,* because it is also a second stage that will help us to recruit possible graduates. But also with a lot of caution for those who will only want to broaden the possibilities in aesthetic medicine*,* because that is not what we are about. We want the nurses to the healthcare system.” [R#2]*

*“There should be such incentives for young people to understand what kind of profession it is*,* the ethos of the profession of a particular professional group should be promoted by individual professional chamber in such a way that people will be willing to approach it.” [R#13]*

### Older and new generations

Participants in our study pointed out that the younger generations’ working styles and needs differ significantly from those of older employees. Young employees appreciate non-wage benefits, the atmosphere at work, the possibility of professional development, the use of their competences for appropriate remuneration and expect their time to be respected, with great emphasis on maintaining work-life balance and mental and physical balance, by providing a multisport card or a relaxation room, yoga, or a fitness zone right in the workplace.

*“Generation “Z” perceives things differently here*,* for them this work-life balance is the most important. We really have to focus on this*,* otherwise soon there will be no people in the entire healthcare system. […] These people really have a completely different approach. These people will not be toiling. […] They will certainly look at the organization of work*,* whether they have such job security*,* as well as legal security” [R#8]*

*“These younger*,* the newest generation*,* which are entering this labour market*,* are indeed increasingly starting to appreciate the work-life-balance. […] We have taken into account a scenario that these people will no longer want to work as intensely as these generations older than them.” [R#15]*

*“It seems that work is better organized in private centers. More of these bonuses*,* these additional things that actually encourage young people. What would encourage young people to work in this particular public sector? A change in working conditions*,* better organization*,* less mess*,* less bureaucracy. More effective use of your competences. Young people often choose a private facility for this reason - medical care*,* some additional fitness cards*,* and that is often why young students of laboratory medicine choose the private sector.” [R#8]*

As our respondents indicate, young doctors have a different system of values, a different way of communicating, they are familiar with modern technology, and at the same time they lack practice and developed interpersonal skills, soft skills, or live communication without the use of instant messengers.

*“Unfortunately*,* we are not able to communicate − 40% of these [adverse] events would not have occurred if it were not for the lack of communication between*,* first*,* members of the therapeutic team and then between the family and the patient.” [R#2]*

Therefore, the market offering courses, training and jobs should take this into account, wanting to encourage taking up work in a place that is not particularly attractive for young graduates. Moreover, younger generations of healthcare professionals, especially doctors, tend to choose easier and more profitable specializations, such as dermatology, aesthetic medicine, and less often choose specializations related to long shifts or hard work in the operating theater.

*“Young people also see that this doctor earns very good money and they also go in this direction [medicine]. The only question is*,* what specializations do they choose? Only the easy ones? I would rather be a dermatologist and have a cultural office*,* just see patients privately. Why should I drive an ambulance*,* in stressful conditions*,* when I can generally earn a lot of money in my office in such a very good and profitable profession*,* specialisation.” [R#13]*

*“Young people are not eager to work in the hospital. Working in a hospital means shifts*,* working in a hospital means responsibility. Among the strong disciplines that are related to surgery*,* the operating room*,* young people try to avoid it. Even if they do a specialization*,* they are also looking for a place where they will have a quiet*,* easy job for good money.” [R#4]*

Additionally, not only educational programs during studies and courses, but also future jobs and employers should adapt their offers to encourage or retain as many young people as possible to work in the healthcare sector.

*“As pharmacists*,* we are going to raise the rank of the profession to show that this profession is not just a job in the pharmacy. Because young people in high school often don’t even know that besides the pharmacy*,* they can still work somewhere.” [R#12]*

*“It’s still about these new competences […] not all healthcare providers use the competences of nurses and that is why a young person joins such a team and sees that there is no possibility of using what she has learned*,* so young people will change their workplace. Everyone wants to be causative*,* and this also involves responsibility.” [R#2]*

*“The younger generation is much more mobile than the older generations.” [R#5]*

### Consequences of actions aimed at mitigating the shortage of healthcare workers and impact on healthcare system

Our respondents also pointed out the consequences of actions aimed at eliminating the shortage of medical staff and, among other things, emphasize the fact that increasing the admission limits for medical studies causes young high school graduates to choose medicine instead of nursing. Therefore, by increasing the number of doctors, we also contribute to reducing the number of new, young nurses, because the medical profession is more attractive.

*“If someone has to choose between the medical or nursing faculties*,* most will choose the medical one because they have different prospects. Maybe more distant*,* but definitely financially much better*,* because talking to some young people who graduated from nursing*,* they graduated because they didn’t get into medical school*,* but they say*,* they already have a profession here*,* but they will try again*,* so we have to deal with this problem.” [R#2]*

Moreover, increasing admission limits and opening medical fields at non-medical universities may result in a decline in the quality of education.

*“When it comes to the education of human resources in the context of quality*,* of course*,* there are such risks that education can have a weaker dimension*,* this staff potentially*,* if there is more*,* will be worse educated.” [R#11]*

Additionally, there are already problems with providing highly qualified teaching staff at medical universities, and with the increased number of universities educating doctors, this problem will increase. Due to the decreasing interest of graduates in selected medical fields and the fact that we want to retain and encourage as many medical professionals as possible, not only doctors, this involves offering higher, attractive salaries that would be competitive with other non-medical professions. As a consequence, the healthcare budget should be increased, but above all, it should be allocated appropriately and transparently.

*“The education system is also competing for talents now. We want more people to get into medicine*,* nursing*,* public health*,* or medical data science. We’re competing for talent with the banking sector*,* the science sector*,* the insurance sector*,* etc. And often with the tech giants*,* who also need that talent. The attractiveness of the health sector has to be high enough that talent wants to go there. The motivations for people to stop working have also changed. That’s another problem here that we can’t forget about.” [R#3]*

Another consequence for the healthcare system is that due to the increased demand and constantly recorded shortages, people are admitted to medical faculties or work without further verification, who, as it later turns out, do not have predispositions to perform this profession, or have certain dysfunctions that should be excluded at the very beginning of the selection process. As a result, state money is invested in the education of people who will never enter the system, will be the cause of patient dissatisfaction and complaints or will leave the profession after working for only a few years.

*“I have a shortage of staff in the Hospital Emergency Department*,* I employ doctors who are not suitable*,* just to have doctors. I have complaints about them.”[R#14]*

*“Nursing departments are really just taking all sorts of people*,* who are not necessarily the ones who should be in nursing. Already during their studies*,* it turns out that these are people who struggle with certain disabilities and health deficits*,* which should be a contraindication to practicing the profession of nurse.” [R#2]*

The consequence of insufficient support and inadequate management, negligence in the case of insufficient staffing - white spots or medical deserts - is that doctors are overloaded, burned out, face increasing mental problems and ultimately change their place of work or resign from the profession.

*“People who have taken part in adverse events*,* medical staff taking part in adverse events or in other traumatising events that develop syndrom similar to post-traumatic stress. These people are more likely to fall out of the profession if they don’t provide psychological care*,* support.” [R#6]*

Moreover, another consequence for the Polish healthcare system resulting from the liberalization of the law regarding the exercise of medical professions by Ukrainians whose country is involved in an armed conflict is the provision of medical services by personnel who do not speak Polish well. This potentially is related to patient complaints due to the inability to communicate smoothly with a medical professional.

*“An example where a doctor who didn’t speak Polish well*,* […] she was fired because there were a lot of complaints […] there were communication barriers somewhere. A doctor is a profession where the patient entrusts their health and the patient requires that this communication should be good. […] so if they can’t get along*,* can’t understand each other and are not sure about their competences*,* then this really doesn’t inspire trust in the patients” [R#5]*

Although a larger number of medical workers deal with a greater burden on our staff who supervise and verify the actions of employees from Ukraine, and in the event of an oversight, it may result in damage to the patient’s health or another undesirable situation with legal consequences.

*“The issue of describing a medical case*,* they say [other Polish-speaking doctors] that they have to correct sometimes the documentation*,* so that there are no mistakes.” [R#14]*

In addition, in many medical facilities, medical workers who should have retired or need rest often continue to work hard because there is no one to replace them.

*“I also have a neurology department and instead of having a full staff*,* I have 3 doctors who are already on their last legs and I wonder what to do during the holidays*,* because they should go on vacation and rest.” [R#14]*

*“Of the 270*,*000 nurses who are actively practicing their professions*,* over 20% are people who have already reached retirement age and are still working […]. We know that these nurses work longer after reaching retirement age and in this situation*,* we also want them to remain in this professional activity.” [R#2]*

The summary of current solutions and those proposed by our respondents to alleviate the shortage of medical personnel in Poland are presented in Table 3.

**Table 3:** Implemented and proposed solutions indicated by respondents

Solutions already implemented	Proposed solutions
increasing admission limits for medical studies	developing and implementing of a long-term plan based on reliable date
opening new medical faculties at non-medical universities	interdisciplinary cooperation of policy makers and researchers from different sectors and fields
legally defined minimum salaries for medical workers	supporting mental health
additional financial and non financial benefits (regionally/locally)	increasing salaries and investment in healthcare workers
scholarships (regionally/locally)	health education and transparent promotion of medical professions from an early age
increasing the scope of competencies of selected groups of medical workers, e.g. nurses, pharmacists, physiotherapists	supporting women in returning to work after maternity leave
various IT solutions, telemedicine, robots, AI	improving working conditions and management
financing or co-financing of postgraduate specialization training	adapting educational and job offers to the needs of new generations
introducing and promoting coordinated care	enabling, promoting and financing of professional development
enabling doctors and nurses from Ukraine to take up employment	enabling work-life balance
introducing new medical and about-medical professions	implementing new guidelines and a clear legal framework according to the use of IT and AI solutions

Source: Authors’ own work

## Discussion

This study aimed to identify possible actions to mitigate and prevent medical staff shortages in Poland. Key findings highlight the urgent need for a long-term strategic plan, improved working conditions, investment in mental health and education, and better use of technology and interprofessional and intersectoral cooperation. Our findings confirmed significant shortages of medical staff in Poland as a whole, as well as in individual regions or specializations. Our respondents indicated solutions that have already been implemented to solve this pressing problem. The most frequently mentioned actions included increasing admission limits to medical studies, increasing the number of institutions educating future medical professionals. In the academic year 2023/2024, 36 institutions educated doctors [31], and the total admission limit for the medical field was 10,289 [31, 32]. For comparison, in 2015, there were 15 universities with a total admission limit of 6,188 places [31]. Additionally, statutory determination of the amount of medical workers’ salaries, increasing the amount of remuneration of individual groups of medical professions, scholarships for people taking up work in unattractive and remote places, and enabling professional development through financing or co-financing specializations were present. It was also emphasized that increasing the limits and number of universities may result in consequences in the form of lower quality of education and insufficient infrastructure conditions and insufficient number of teaching staff. Respondents also pointed to difficulties and problems in the healthcare system resulting from deficits and specifics of individual medical professions and indicated inadequate management, lack of a well-thought-out, data-based and long-term health policy plan. The views of our respondents also coincide with the views of Polish researchers who indicate that despite the already introduced salary increases [33], their level is still unsatisfactory with significant disparities, there is low professional prestige, high workload, professional burnout, high level of stress and difficulty in combining professional work and family life [34]. A particularly acute issue is the shortage of nurses. According to data from the Central Register of Nurses and Midwives, as of January 2023, there were 315,670 registered nurses in Poland, of whom only approximately 74% were actively employed in the profession. This indicates that over 25% of registered nurses are not currently practicing, highlighting a significant workforce gap [35]. Additionally, these staff shortages contribute significantly to limited access to healthcare services. According to the “WHC BAROMETER. Poles in queues. Report on changes in access to guaranteed health care services in Poland”, in November 2022, the waiting time for a single health service was 3.6 months, while to obtain the advice of a specialist, the waiting time was on average 4.1 months. The authors of the report indicate that the problem of limited access to specialists and long waiting times for services referred by primary care physicians is clearly visible, which translates into a prolonged and multi-stage treatment path [36]. Although Poland has a universal health coverage system and citizens are granted equal access to the publicly-funded healthcare system, many Poles rely on private sector healthcare services due to low availability and long waiting times. The Public Opinion Research Centre’s (CBOS) 2023 Research Announcement indicated that only one-quarter of respondents used medical services exclusively within the framework of public health insurance in the six months prior to the survey (24%), and one-tenth (11%) used services financed independently or available under a subscription or policy. The largest group accounting for as much as 51% of Poles used both private and public healthcare services. The reason of using private services cited by eight out of ten respondents (79%) - was invariably shorter waiting times for appointments [37].

–The proposals for solutions to the problem of the shortage of medical personnel indicated by our respondents and other researchers, concern the development and implementation of a long-term plan - education, financing, management of healthcare workers. This requires a thorough evaluation, collection of reliable data [4], and above all, the involvement of many sectors and interdisciplinary cooperation in the real implementation of science-based solutions [1, 2, 8–10, 13]. Our respondents indicate increased investment in healthcare workers, but also emphasize the importance of transparency. Additionally, European researchers indicate that transparent salaries and equalization of the gender pay gap are actions that need to be taken, because almost three-quarters of healthcare workers in the world are women, and among nurses and midwives it is as much as 97% [19, 20, 38]. The researchers and participants of our study unanimously emphasize that to retain current employees and encourage new ones to work in the healthcare sector, it is necessary to improve working conditions, support mental health [11, 12, 39], enable professional development and support women in returning to work after maternity leave, creating opportunities for them to combine professional and family responsibilities [9, 17, 39–41]. Findings of our study confirmed that simply adding new jobs or increasing limits for medical studies, with inappropriate work organization, lack of respect and poor management will not alleviate the shortage [17, 32, 38].

The education plan for future healthcare workers is essential to reduce the shortage of medical personnel; an adequate number of medical professionals and their competences must meet the needs of an ageing society [9, 41–44]. Due to the changes, both technological and cultural, in terms of work style and environment, as well as the needs of employees, evaluation programs should include various representatives of practice in order to constantly verify the adequacy of the program content and teaching methods [45]. Our respondents suggested that attention should be paid to the teaching staff, that it should be composed primarily of professionally active people, so that only theoretical or historical content is not taught. In addition, real cooperation between professions should be included in teaching, not simulations with role-playing during classes, but practical cooperation using the latest technologies preparing for real work in teams [15, 41–44].

In addition, task-shifting and skill-mix are effective and popular methods in many countries to reduce the shortage of medical staff [46, 47]. Increasing the scope of competences of other groups of healthcare professionals allows for reducing the workload of doctors, who can then deal with more difficult cases [43, 46–48]. Both our respondents and other Polish researchers emphasize that despite the expansion of competences of nurses, midwives and pharmacists [49], their potential is still not fully utilized [50–52]. Our results are consistent with current national discussions within the nursing profession in Poland, where there is an increasing emphasis on expanding the role of nurses in primary care and implementing advanced practice roles. There is a growing awareness that professional development must be accompanied by systemic change. Promotion of the nursing and midwifery profession, motivation, engagement and encouragement of leadership are essential. Empowering nurses to practice to the full extent of their competences– especially in outpatient settings– could alleviate workforce shortages and improve continuity of care, especially in underserved regions [51]. Additionally, the problem in Poland is the underestimation of the role and lack of knowledge about the broad competences of a family doctor; patients often force GPs to refer patients to specialists because they believe that they will receive better care, which generates additional visits and costs [53]. This may also be due to the fact that public trust in the healthcare system is low [53], and that patients are becoming increasingly self-conscious and do not feel fully involved in the treatment process [52]. Many Polish doctors have insufficient ethical and interpersonal competences to conduct dialogue and joint decision-making, there is a lack of space for empowering patients and their families [52–54]. Doctors are afraid of patients’ expectations and, as our respondents indicated, they also feel fear of lawsuits from patients, which additionally causes stress and can lead to burnout and, consequently, a change of profession. Therefore, support, stress management practices and trainings should be implemented to prevent burnout and deepening the deficit [9, 11].

### Limitations

Our study is not free from limitations. First, the study results present opinions of Polish key stakeholders and refer to the Polish healthcare system, which cannot be generalized to other countries whose healthcare delivery systems are determined by different political situations and are organized in terms of legal, financial and clinical aspects in distinct and specific ways. However, analyzing Polish problems and possible solutions can be a source of inspiration and knowledge for researchers and decision-makers from other countries. Secondly, although the topics and methodology of conducting interviews seemed consistent among all study participants, there is a chance that during data collection and analysis, some aspects of the statements may have been misinterpreted or underestimated. This is because the perception of the interlocutors may be influenced by the participants’ personal experiences and working conditions. To eliminate the excessive influence of personal views, we included representatives of all healthcare professions’ chambers and various healthcare institutions in the study, which allows us to provide a broader picture of this problem. Finally, while the sample size may appear modest, it is in line with established standards for qualitative research involving in-depth interviews with strategically selected experts. Previous literature suggests that even a small number of interviews — as few as six — may be sufficient to identify the principal thematic patterns in qualitative research [25]. In our case, saturation was reached after 12 interviews, and all 15 originally planned interviews were conducted to ensure analytical richness and prevent premature closure.

## Conclusions

To alleviate the shortage of healthcare workers in Poland, a long-term, comprehensive plan for education, financing, and workforce management based on reliable data should be developed and implemented. Intersectoral collaboration and evidence-based planning are essential. Improving working conditions, supporting mental health, and creating appropriate facilities for women are actions that should be implemented. Enabling work-life balance, ensuring adequate remuneration and providing development opportunities are also crucial. Promotion of medical professions and transparent career pathways in healthcare should be reinforced. Further research is needed to assess the long-term impact of policy changes on workforce distribution and the effectiveness of interventions, such as additional stipends, task shifting, and IT solutions.

## Supplementary Information

Supplementary Material 1. Appendix 1. In-depth interview scenario

Supplementary Material 2. Appendix 2. The Consolidated Criteria for Reporting Qualitative Research (COREQ) protocol

## Data Availability

The datasets presented in this article are not publicly available, as the full content of our respondents’ answers remains undisclosed to ensure anonymity. Requests for access to the datasets should be directed to KM, kamila.michalska@doctoral.uj.edu.pl.
