# Supplementary material for: Addressing the health workforce crisis in Poland from the key stakeholders’ perspectives – a qualitative study
Source: BMC Health Serv Res. 2025 Aug 22;25:1121. doi: 10.1186/s12913-025-13150-5 (PMC12372184; doi:10.1186/s12913-025-13150-5)
Supplement: Supplementary file 1 — Supplementary Material 1. Appendix 1. In-depth interview scenario [file 12913_2025_13150_MOESM1_ESM.docx]

***Addressing the health workforce crisis in Poland from the key stakeholders’ perspectives – qualitative study.***

**Authors:** Kamila Michalska^1,2^, Alicja Domagała^1^

1- Institute of Public Health, Faculty of Health Sciences, Jagiellonian University Medical College, Krakow, Poland

2 - Doctoral School of Medical and Health Sciences, Jagiellonian University Medical College, Krakow, Poland

Appendix 1.

In-depth interview scenario

1. How do you assess the current shortage of medical personnel in Poland and its impact on the healthcare system?

2. What actions or tools are currently used to reduce the effects of medical personnel shortages in Poland?

3. What are your suggestions for effective actions and tools that can be used to solve the problem of medical personnel shortages in Poland?

4. What are the main difficulties you encounter in connection with the current shortage of medical personnel?

5. Do you know of examples of effective actions or tools that eliminate the effects of medical personnel shortages in other countries that could be implemented in Poland?

6. What is your opinion on the role of medical education and training in combating medical personnel shortages? Are there specific areas where investment in medical education or training should be increased?

7. How do you assess the current government policies and regulations regarding the employment and migration of medical workers in the context of eliminating medical personnel shortages and their effects?

8. What do you think are the possibilities and prospects for cooperation between the public and private sectors to effectively eliminate the shortage of medical personnel in Poland? What are the potential benefits and challenges associated with such cooperation?

9. How are new IT solutions currently used to eliminate the effects of shortages? What other solutions can still be implemented in this area?

10. What do you think are the potential consequences or challenges associated with introducing actions or tools aimed at eliminating the effects of medical personnel shortages in Poland?
