# Supplementary material for: Addressing the health workforce crisis in Poland from the key stakeholders’ perspectives – a qualitative study
Source: BMC Health Serv Res. 2025 Aug 22;25:1121. doi: 10.1186/s12913-025-13150-5 (PMC12372184; doi:10.1186/s12913-025-13150-5)
Supplement: Supplementary file 2 — Supplementary Material 2. Appendix 2. The Consolidated Criteria for Reporting Qualitative Research (COREQ) protocol [file 12913_2025_13150_MOESM2_ESM.docx]

***Addressing the health workforce crisis in Poland from the key stakeholders’ perspectives – qualitative study.***

**Authors:** Kamila Michalska^1,2^, Alicja Domagała^1^

1- Institute of Public Health, Faculty of Health Sciences, Jagiellonian University Medical College, Krakow, Poland

2 - Doctoral School of Medical and Health Sciences, Jagiellonian University Medical College, Krakow, Poland

**Appendix 2.**

**Consolidated criteria for reporting qualitative studies (COREQ): 32-item checklist**

*Developed from: Tong A, Sainsbury P, Craig J. Consolidated criteria for reporting qualitative research (COREQ): a 32-item checklist for interviews and focus groups. International Journal for Quality in Health Care. 2007. Volume 19, Number 6: pp. 349 – 357.*

**YOU MUST PROVIDE A RESPONSE FOR ALL ITEMS. ENTER N/A IF NOT APPLICABLE**

| **No. Item** | **Guide questions/description** | **Response** |
| --- | --- | --- |
| **Domain 1: Research team and reﬂexivity** |  |  |
| *Personal Characteristics* |  |  |
| 1. Inter viewer/facilitator | Which author/s conducted the interview? | Kamila Michalska |
| 2. Credentials | What were the researcher’s credentials? E.g. PhD, MD | Kamila Michalska, MSc, PhD Candidate |
| 3. Occupation | What was their occupation at the time of the study? | Kamila Michalska, PhD Student at the Doctoral School of Medicine and Health Sciences |
| 4. Gender | Was the researcher male or female? | Female |
| 5. Experience and training | What experience or training did the researcher have? | Extensive qualitative experience through education and additional training prior the study. |
| *Relationship with participants* |  |  |
| 6. Relationship established | Was a relationship established prior to study commencement? | No relationship with interviewers prior to the study. |
| 7. Participant knowledge of the interviewer | What did the participants know about the researcher? e.g. personal goals, reasons for doing the research | Prior to each interview, respondents were informed about the overview of the study and the objectives of the research (exploration of the health workforce shortages in Poland, methods and tools to mitigate the effects of them). |
| 8. Interviewer characteristics | What characteristics were reported about the interviewer/facilitator? e.g. Bias, assumptions, reasons and interests in the research topic | Interviewer is PhD candidate and a involved in the study on behalf of the Institute of Public Health. Participants were informed that the study protocol was approved by the Bioethical Committee of the Jagiellonian University. |
| **Domain 2: study design** |  |  |
| *Theoretical framework* |  |  |
| 9. Methodological orientation and Theory | What methodological orientation was stated to underpin the study? e.g. grounded theory, discourse analysis, ethnography, phenomenology, content analysis | In this qualitative study we used content analysis. |
| *Participant selection* |  |  |
| 10. Sampling | How were participants selected? e.g. purposive, convenience, consecutive, snowball | Purposive sampling was used. Participants were selected based on their knowledge and experience of the topic and active membership in health professionals’ association. The participation in the study was voluntary. |
| 11. Method of approach | How were participants approached? e.g. face-to-face, telephone, mail, email | Participants were approached through telephone or email. |
| 12. Sample size | How many participants were in the study? | 15 participants were interviewed. |
| 13. Non-participation | How many people refused to participate or dropped out? Reasons? | No one refused to participate. |
| *Setting* |  |  |
| 14. Setting of data collection | Where was the data collected? e.g. home, clinic, workplace | The interviews were conducted and data was collected using the on-line MS Teams platform. |
| 15. Presence of non-participants | Was anyone else present besides the participants and researchers? | No. Only participant and interviewer were present. |
| 16. Description of sample | What are the important characteristics of the sample? e.g. demographic data, date | Representatives of Polish key stakeholders working in healthcare sector, including: doctor, nurse, physiotherapist, lawyer, public health specialist and paramedic. Seven were male and eight female. |
| *Data collection* |  |  |
| 17. Interview guide | Were questions, prompts, guides provided by the authors? Was it pilot tested? | Yes, the interview guide consisted of 10 questions.  No, the interview guide was not pilot tested. |
| 18. Repeat interviews | Were repeat interviews carried out? If yes, how many? | None |
| 19. Audio/visual recording | Did the research use audio or visual recording to collect the data? | All the interviews were audio recorded and transcribed verbatim. In addition, 11 of the 15 interviews were also video recorded. |
| 20. Field notes | Were ﬁeld notes made during and/or after the interview? | No |
| 21. Duration | What was the duration of the interviews or focus group? | The interviews lasted on average 60 min. (min. 33 min. and max. 89 min). |
| 22. Data saturation | Was data saturation discussed? | Yes |
| 23. Transcripts returned | Were transcripts returned to participants for comment and/or correction? | Yes, but only to one participant, because she requested it. |
| **Domain 3: analysis and ﬁndings** |  |  |
| *Data analysis* |  |  |
| 24. Number of data coders | How many data coders coded the data? | 1 |
| 25. Description of the coding tree | Did authors provide a description of the coding tree? | No |
| 26. Derivation of themes | Were themes identiﬁed in advance or derived from the data? | Themes were identified from the interview guide topics and then further finalized from collected data |
| 27. Software | What software, if applicable, was used to manage the data? | NVivo |
| 28. Participant checking | Did participants provide feedback on the ﬁndings? | No |
| *Reporting* |  |  |
| 29. Quotations presented | Were participant quotations presented to illustrate the themes/ﬁndings? Was each quotation identiﬁed? e.g. participant number | Yes, see section “Results”. In each quotation we provided participants’ numbers. |
| 30. Data and ﬁndings consistent | Was there consistency between the data presented and the ﬁndings? | Yes |
| 31. Clarity of major themes | Were major themes clearly presented in the ﬁndings? | Yes |
| 32. Clarity of minor themes | Is there a description of diverse cases or discussion of minor themes? | Yes |

*Once you have completed this checklist, please save a copy and upload it as part of your submission. When requested to do so as part of the upload process, please select the file type: Checklist. You will NOT be able to proceed with submission unless the checklist has been uploaded.*
